# Pseudo-progression with osimertinib after definitive chemoradiation in unresectable epidermal growth factor receptor mutation positive of stage III non-small cell lung cancer: A case report

**DOI:** 10.3389/fonc.2022.971192

**Published:** 2022-08-30

**Authors:** Fei Ren, Yao Wang, Yongsheng Gao, Xiangjiao Meng

**Affiliations:** ^1^ Department of Radiation Oncology, Shandong Cancer Hospital and Institute, Shandong First Medical University and Shandong Academy of Medical Sciences, Jinan, China; ^2^ Department of Pathology, Shandong Cancer Hospital and Institute, Shandong First Medical University and Shandong Academy of Medical Sciences, Jinan, China; ^3^ Department of Radiation Oncology, Shandong Cancer Hospital and Institute Affiliated to Shandong University, Jinan, China

**Keywords:** pseudoprogression, osimertinib, EGFR mutation, NSCLC, case report

## Abstract

Epidermal growth factor receptor tyrosine kinase inhibitors (EGFR TKIs) have been widely used in the treatment of locally advanced non-small cell lung cancer (NSCLC). The phenomenon of pseudoprogression in targeted therapy in EGFR-mutation NSCLC patients is rare. Here, we reported an EGFR-mutation-positive lung adenocarcinoma patient who was admitted to a hospital for cough and chest distress accompanied by shortness of breath. He underwent four cycles of chemotherapy with pemetrexed combined with carboplatin and concurrent radiotherapy in the third and fourth cycles. Then, he was treated by osimertinib maintenance therapy. After 11.5 months of osimertinib treatment, he was assessed to progressive disease by computed tomography. He underwent fiber bronchoscopy, and the biopsy pathology showed extensive necrosis without tumor cells. Until now, the patient has continued on osimertinib for 7 months without relapse or metastasis. As far as we know, we are the first to report pseudoprogression in osimertinib maintenance after definitive chemoradiation. This study reminds the clinicians to distinguish pseudoprogression from osimertinib-induced progression and avoid abandoning effective treatments.

## Introduction

At present, immune checkpoint inhibitors and targeted drugs have become widely used in locally advanced non-small cell lung cancer (NSCLC) (1, 2). Pseudoprogression is a special phenomenon, which is manifested by increased volume or appearance of new lesions with subsequent narrowing of the mass (3). The overall rate of pseudoprogression in immunotherapy was 5.0% (95% CI: 3.4%, 6.7%) in NSCLC patients (4). Two case reports showed three ALK-positive NSCLC patients who developed pseudoprogression by magnetic resonance imaging (MRI) after alectinib treatment (5, 6). Data are scarce on pseudoprogression in epidermal growth factor receptor (EGFR) mutation NSCLC patients associated with targeted drug monotherapy. In this study, we reported a patient with lung adenocarcinoma who developed pseudoprogression in osimertinib maintenance after definitive chemoradiation.

## Case presentation

**Figure 1:**
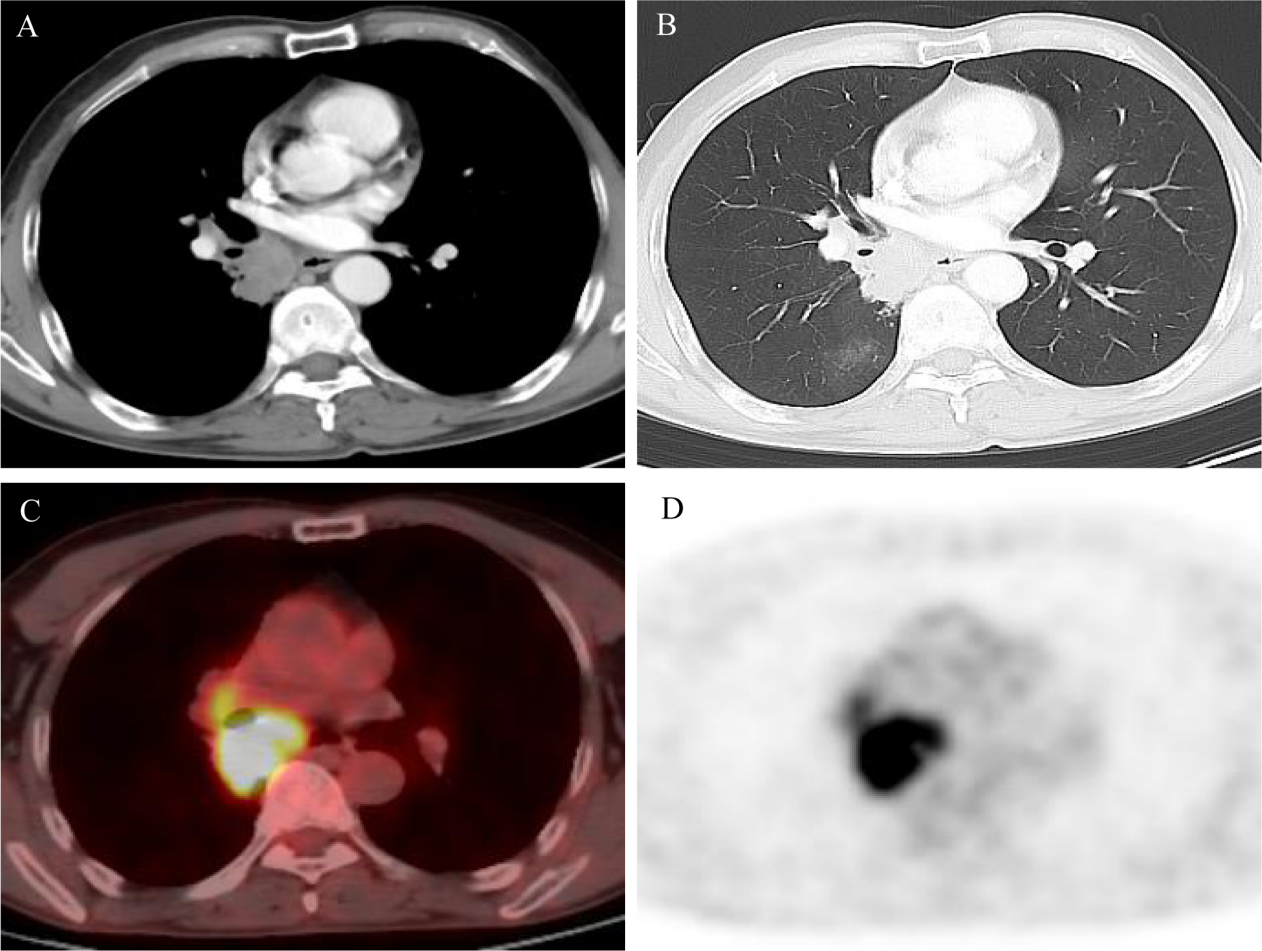
**(A, B)** Computed tomography (CT) scan at diagnosis showed a lung mass in the right lower lobe and mediastinum; **(C, D)** positron emission tomography/computed tomography (PET/CT) scan at diagnosis showed high fluorodeoxyglucose avidity.

**Figure 2:**
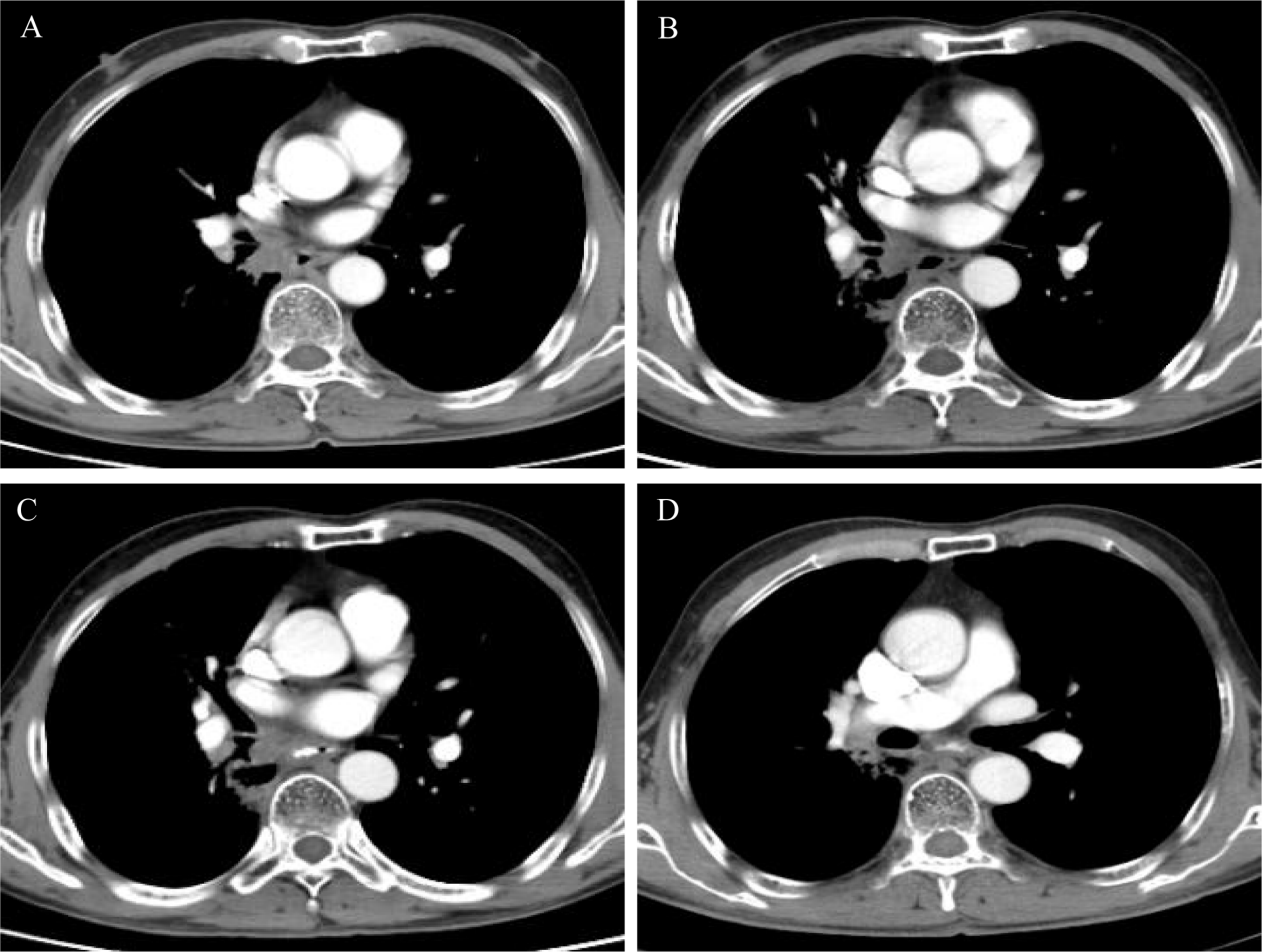
**(A)** Computed tomography (CT) scan after chemoradiation showed partial response; **(B–D)** CT scan showed stable disease during osimertinib treatment.

**Figure 3:**
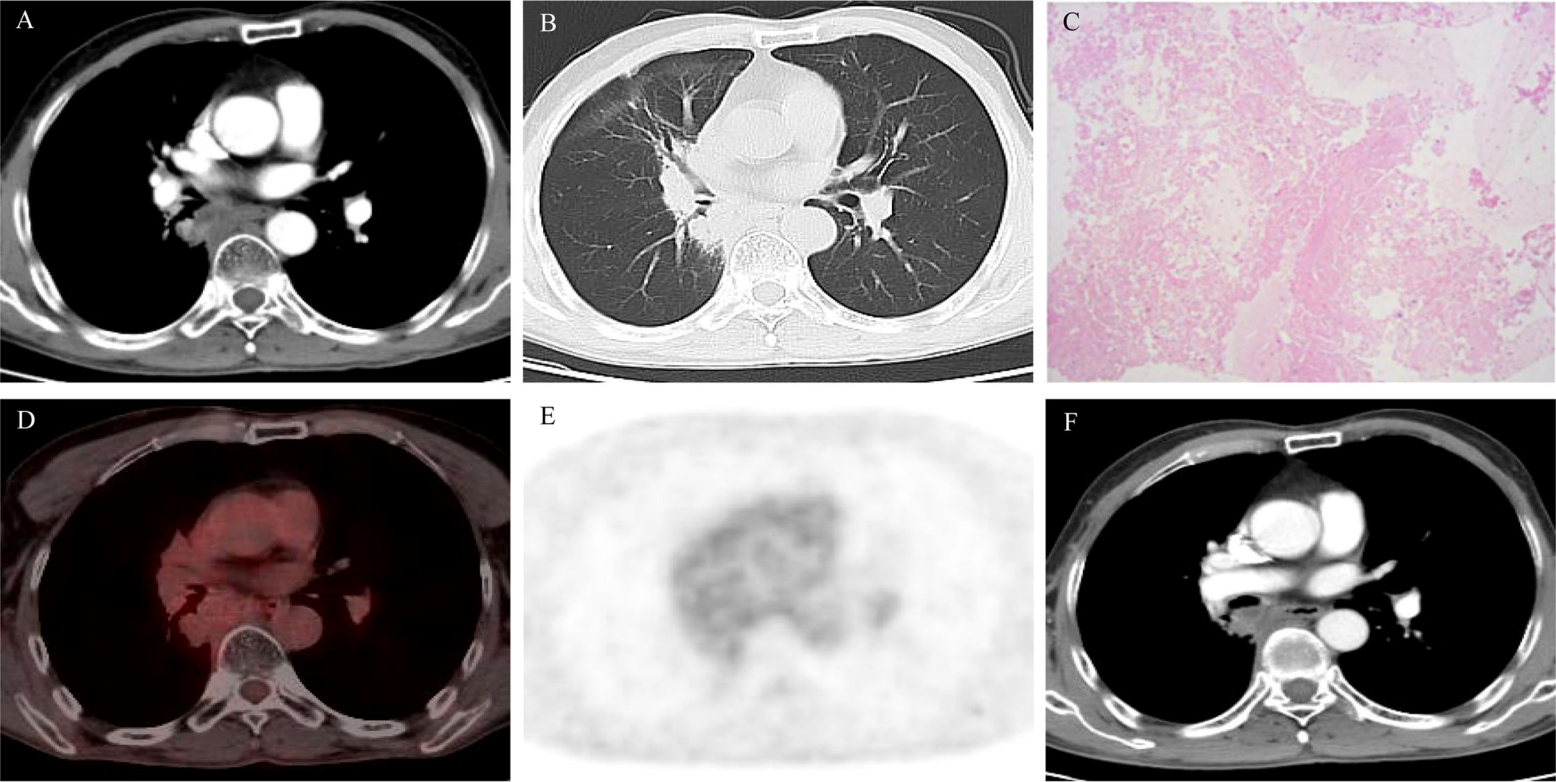
**(A, B)** Computed tomography (CT) scan after 11.5 months of osimertinib therapy showed enlarged primary mass; **(C)** hematoxylin and eosin (H&E) stain of the mediastinal biopsy tissue sample demonstrating extensive necrosis without tumor cells; **(D, E)** PET/CT scan after 11.5 months of osimertinib therapy showed no fluorodeoxyglucose avidity; **(F)** CT scan after 18.5 months of osimertinib treatment showed no relapse or metastasis.

**Figure 4:**
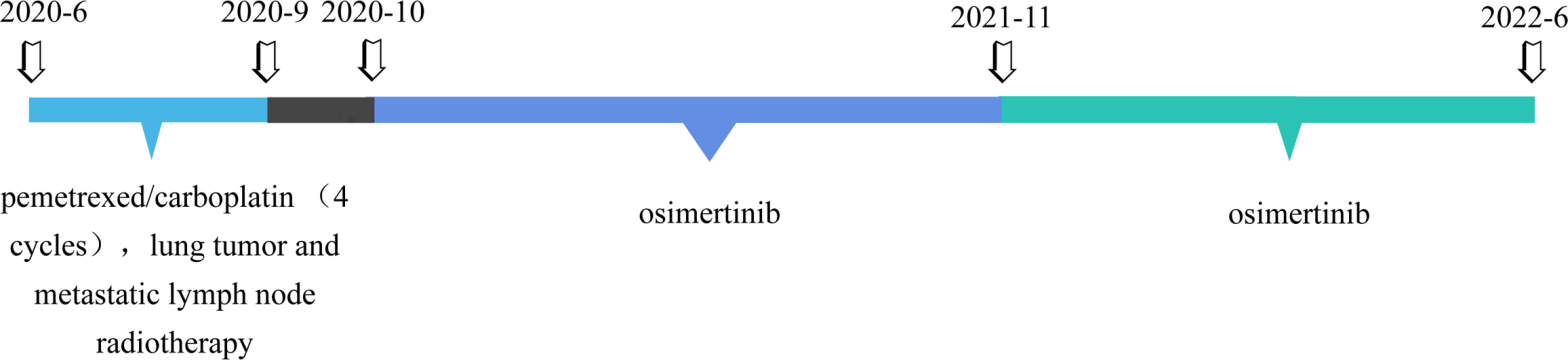
Timeline of the interventions and outcomes.

This patient is a 65-year-old man who was hospitalized for cough and chest distress accompanied by shortness of breath. He was a previously healthy never smoker and had no family history of tumors. No significant abnormalities were observed on physical examination. Carcinoembryonic antigen (CEA) and cytokeratin 19 fragments were high and procalcitonin was in the normal range. Chest computed tomography (CT) and positron emission tomography/computed tomography (PET/CT) showed the right lower lobe lung cancer (35 mm×60 mm) (SUVmax = 14.1) with the right hilar and mediastinal lymph node metastasis (**Figures 1A** **–** **2D**). Cranial-enhanced MRI showed no brain metastases. He underwent fiber bronchoscopy, and the biopsy pathology showed adenocarcinoma. The gene mutation test indicated EGFR exon 21 L858R mutation. He was diagnosed with EGFR-mutation-positive stage IIIB (cT3N2M0) lung adenocarcinoma. He underwent four cycles of chemotherapy with pemetrexed (800 mg on day 1, every 3 weeks) combined with carboplatin (500 mg on day 2, every 3 weeks) and concurrent lung tumor and metastatic lymph node radiotherapy (RT) (60 Gy/30 fractions) in the third and fourth cycles. After chemoradiation, reexamination CT showed partial response (**Figure 2A**). Then, he was enrolled in a clinical trial, which evaluates the efficacy and safety of osimertinib maintenance after definitive chemoradiation in unresectable EGFR mutation positive stage III NSCLC (LAURA trial) (7). During osimertinib treatment, the therapeutic effect evaluated by CT was always stable disease (SD) (**Figures 2B–D**). After 11.5 months of osimertinib treatment, he reviewed the chest enhanced CT showing the mass enlarged, which was inhomogeneous reinforcement (**Figures 3A, B**). He was assessed as having progressive disease (PD) according to the evaluation criteria of RECIST 1.1. The increased CT value after enhanced CT is 23 hounsfield unit (HU), whereas the increased value is 33 HU before osimertinib. Tumor markers were in the normal range. He underwent fiber bronchoscopy, and no tumor cells were detected by the aspiration cytology of the mediastinal lumps; the biopsy pathology showed extensive necrosis without tumor cells (**Figure 3C**). PET/CT showed no high fluorodeoxyglucose avidity (SUVmax = 3.4) to avoid inaccurate puncture due to tumor heterogeneity (**Figures 3D, E**). Therefore, the therapeutic effect was SD. Now, the patient has continued on osimertinib for 7 months without relapse or metastasis (**Figure 3F**). The overall course of treatment is shown in **Figure 4**, and the compliance of this patient was quite high.

## Discussion

In our study, we are the first to report pseudoprogression in osimertinib maintenance after definitive chemoradiation. However, it is not clear whether the necrosis was caused by RT or osimertinib treatment or both. Radiation therapy can cause vascular endothelial damage, leading to the increase in vascular permeability and the release of some pro-inflammatory cytokines, concomitant with the upregulation of vascular endothelial growth factor (VEGF), and ultimately facilitating7 radioactive injury lesion expansion (8). A case report showed a stage IIIA adenocarcinoma patient who developed pseudoprogression at 4 months after stereotactic body radiotherapy (SBRT) (9). Michael C Stauder et al. reported that the pseudoprogression occurred at 12 months after stereotactic ablative RT (10). Another study showed that the median time for developing an enlarged lesion in the area of SBRT was 12 months in patients without tumor recurrence (11). This suggests that the possibility of radionecrosis can occur at several months to one year after RT.

Osimertinib, a third-generation EGFR tyrosine kinase inhibitor (TKI), is now the first-line treatment for EGFR-mutation-positive advanced NSCLC (1, 2). VEGF and EGFR have many overlapping and parallel downstream pathways, and the activation of EGFR can upregulate the expression of VEGF and VEGFR and facilitate VEGFR activating (12). The combination therapy of anti-angiogenic drugs and EGFR-TKIs can simultaneously block the VEGFR/EGFR pathway, which has synergistic effects to enhance the antitumor effect (13, 14). In a clinical trial, osimertinib combined with bevacizumab showed that the overall response rate was 80% (95% CI, 67–91%), and the median progression free survival (PFS) was 19 months (95% CI, 15–24 months) (15). However, VEGF expression is affected by many factors, including cytokines (e.g., tumor necrosis factor-α [TNF-α], transforming growth factor-β [TGF-β], EGF, fibroblast cytokines, and interleukin-1), oncogene expression (e.g., EGFR, erbB2/human EGFR 2 [HER2], ras, and src), and hypoxia (16). The EGF/EGFR pathway is one of the many factors that regulate VEGF expression. EGFR inhibition alone does not block VEGF, thereby allowing tumor angiogenesis (17). We cannot make the certain claim that pseudo-progression was due to endothelial injury.

The patient’s enhanced CT demonstrated the mediastinum invasion by lung tumor with unclear pericardial demarcation. He was diagnosed as EGFR-mutation-positive unresectable locally advanced lung adenocarcinoma. The standard treatment of unresectable locally advanced NSCLC is concurrent chemoradiotherapy (CCRT) (18). In a retrospective study of EGFR mutation positive of stage IIIB lung adenocarcinoma, there were no statistically significant differences in the 5-year overall survival (OS) rates between TKIs and CCRT groups (30% vs. 26%) (19). Phase I trial of erlotinib combined with CCRT and the SWOG S0023 trial also failed to prove any benefit of TKI addition (20, 21). In unresectable EGFR-mutated positive stage III NSCLC patients, the initial results indicated that the median PFS of EGFR TKI and CRT was significantly longer than that of CRT alone (26.1 months vs. 6.9 months, log-rank *P* = 0.023) (22). In the RECEL trial, compared with etoposide/cisplatin concurrent RT, the median PFS with erlotinib concurrent RT was significantly longer (24.5 vs. 9.0 months [*P* < 0.001]) (23). The LAURA trial will assess the efficacy of osimertinib after definitive chemoradiation in EGFR mutation NSCLC patients (7).

In conclusion, this is the first report of pseudoprogression in osimertinib maintenance after definitive chemoradiation. The mechanism of pseudoprogression after osimertinib treatment is still unclear, but the significance of this study suggests that clinicians should not easily interrupt osimertinib treatment when imaging progression occurs during osimertinib, and further determine pathological examination and PET-CT before making decisions. There are very few relevant studies about the mechanism and possibility of whether EGFR-TKI targeted therapy combined with RT increases pseudoprogression compared with RT alone; it deserves further exploration and discussion.

## Data availability statement

The original contributions presented in the study are included in the article/supplementary material. Further inquiries can be directed to the corresponding authors.

## Ethics statement

The studies involving human participants were reviewed and approved by the Ethics Committee of Shandong Cancer Hospital and Institute. The patients/participants provided their written informed consent to participate in this study.

## Author contributions

FR performed the literature search and wrote the original draft, FR and YW collected and analyzed the data, and XM and YG revised the manuscript critically for important scientific content. All authors contributed to the article and approved the submitted version.

## Funding

This work was supported by CSCO-Haosen Cancer Research Fund (No. Y-HS202102-0089).

## Acknowledgments

We thank the patient and his family for giving consent to this case report.

## Conflict of interest

The authors declare that the research was conducted in the absence of any commercial or financial relationships that could be construed as a potential conflict of interest.

## Publisher’s note

All claims expressed in this article are solely those of the authors and do not necessarily represent those of their affiliated organizations, or those of the publisher, the editors and the reviewers. Any product that may be evaluated in this article, or claim that may be made by its manufacturer, is not guaranteed or endorsed by the publisher.
